# The SAR analysis of dietary polyphenols and their antagonistic effects on bortezomib at physiological concentrations

**DOI:** 10.3389/fphar.2024.1403424

**Published:** 2024-07-25

**Authors:** Tran Tran Thi Van, Hsun-Shuo Chang, Ho-Cheng Wu, Chung-Kuang Lu, Hui-Chi Huang, Michal Korinek, Hui-Hua Hsiao, Chia-Hung Yen

**Affiliations:** ^1^ Graduate Institute of Natural Products, Kaohsiung Medical University, Kaohsiung, Taiwan; ^2^ Drug Development and Value Creation Research Center, Kaohsiung Medical University, Kaohsiung, Taiwan; ^3^ School of Pharmacy, College of Pharmacy, Kaohsiung Medical University, Kaohsiung, Taiwan; ^4^ Department of Medical Research, Kaohsiung Medical University Hospital, Kaohsiung, Taiwan; ^5^ Graduate Institute of Pharmacognosy, College of Pharmacy, Taipei Medical University, Taipei, Taiwan; ^6^ National Research Institute of Chinese Medicine, Taipei, Taiwan; ^7^ Department of Life Sciences and Institute of Genome Sciences, College of Life Science, National Yang Ming Chiao Tung University, Taipei, Taiwan; ^8^ School of Chinese Medicine and Graduate Institute of Chinese Medicine, China Medical University, Taichung, Taiwan; ^9^ Division of Hematology and Oncology, Department of Internal Medicine, Kaohsiung Medical University Hospital, Kaohsiung, Taiwan; ^10^ Faculty of Medicine, Kaohsiung Medical University, Kaohsiung, Taiwan

**Keywords:** multiple myeloma, boronic acid-based proteasome inhibitor, bortezomib, polyphenol, vicinal diol moieties, physiological concentrations

## Abstract

**Background:** Bortezomib (BTZ), a primary treatment for MM, but its effectiveness can be reduced by interactions with vicinal diol moieties (VDMs) in polyphenols. Despite this, it’s debated whether BTZ therapy necessitates avoiding polyphenol-rich products, given the low bioavailability of polyphenols. Additionally, it remains unclear whether the structure of polyphenols contributes to their BTZ antagonism. Therefore, our study aims to unravel the structure-activity relationship of dietary polyphenols and their BTZ antagonism at daily diet-achievable physiological concentrations.

**Methods:** We assessed the antagonistic effects of 25 polyphenols against BTZ using cell viability assays in RPMI 8226 cells. ChemGPS-NP helped analyze the structural similarity. Additionally, long-term cytotoxicity assays evaluated these effects at physiologically relevant concentrations.

**Results:** By cell viability assays, we found a positive correlation between the number of VDMs in gallotannins and their BTZ antagonism. Moreover, the origin and configuration of VDMs, rather than the total VDM concentration, play a pivotal role in the combined antagonistic effects against BTZ in gallotannins. Additionally, ChemGPS-NP analysis indicated that the aromaticity and C-3 hydroxyl group in flavonoids’ C-rings enhance their BTZ antagonism. Finally, long-term cytotoxicity assays reveal that gallic acid (GA), epigallocatechin (EGC), and epigallocatechin gallate (EGCG), at their physiological concentrations—attainable through tea consumption—significantly and synergistically antagonize BTZ.

**Conclusion:** Due to the potential for these polyphenols to reduce the effectiveness of BTZ, it is advisable for MM patients undergoing BTZ treatment to reduce their consumption of foods high in VDM-containing polyphenols.

## 1 Introduction

Multiple myeloma (MM) is a cancer of plasma cells. From 1990 to 2019, global MM incident and death cases more than doubled (Zhou et al., 2021). The introduction of bortezomib (BTZ, Trade name Velcade®), a novel first‐in‐class proteasome inhibitor (PI), has been a significant breakthrough in the treatment of MM. BTZ blocks proteasome’s chymotrypsin-like activity by forming a covalent bond between its boronic acid group and the hydroxy group of the β5 subunit N-terminal threonine of the 20S proteasome (Boike et al., 2022). Through binding to the proteasome, BTZ causes abnormal protein accumulation, leading to ER stress and apoptosis. Myeloma cells, with their high immunoglobulin production and protein synthesis rates, depend more on proteasome function to prevent overload compared to normal cells. Consequently, MM cells are more sensitive to BTZ. Despite the advancements in cancer therapies, drug resistance remains a significant challenge in the management of cancer, including MM (Ahmad et al., 2023; Kozalak et al., 2023). Until now, MM has remained incurable. Therefore, understanding the underlying mechanism caused resistance to BTZ is important for improving MM treatment.

Polyphenols, a large family of natural compounds with multiple phenol groups, are abundant in fruits, vegetables, herbs, spices, tea, dark chocolate, and wine. They offer various health benefits, including anticancer properties, making polyphenol-rich foods and supplements beneficial for health and disease prevention. However, studies reveal that epigallocatechin gallate (EGCG)´s vicinal diol groups can react with the boronic acid in BTZ, thereby inhibiting its anticancer effects in MM cells (Golden et al., 2009; Kim et al., 2009; Jia and Liu, 2013). In a previous study, the antagonistic effects of EGCG (administered at 25 and 50 mg/kg/day via intragastric administration) against BTZ were demonstrated in a mouse xenograft model (Golden et al., 2009). It is important to note that it is highly unlikely for humans to intake such a large amount of EGCG (approximately 3,500 mg/person/day), leading to the conclusion that dietary intake of EGCG should not be high enough to exert noticeable antagonistic effects against BTZ. However, it is interesting to note that another previous study proved the peak plasma levels of EGCG in mice were 0.28 ± 0.08 μM (a concentration close to physiological levels in humans) after intragastric administration of EGCG at 75 mg/kg (Lambert et al., 2003). These results indicated that mice require a much higher EGCG intake to reach plasma concentrations comparable to humans. These findings also suggested that sub-physiological concentrations of EGCG may be sufficient for antagonizing BTZ’s anti-MM effect. However, higher concentration ranges (1–100 μM) of polyphenols were used to evaluate their antagonistic effects against BTZ in MM cell models in previous reports. It remains unclear whether the antagonistic effects of polyphenols at physiological concentrations against BTZ at approximately the average blood concentration can be observed in MM cell models. Furthermore, the potential synergistic antagonistic effects of various polyphenols consumed through a daily diet have not been thoroughly investigated. This study aims to determine whether dietary polyphenols can antagonize BTZ at physiological concentrations and if they exhibit synergistic antagonistic effects against BTZ. Additionally, how the structural features of dietary polyphenols contribute to antagonizing BTZ remains an important yet underexplored area. Our research seeks to elucidate the relationship between the structure of polyphenols and their antagonistic effects.

## 2 Methods and materials

### 2.1 Materials and chemicals

The bortezomib (BTZ) was obtained from Sigma-Aldrich (St. Louis, MO, United States). Ixazomib (#HY-10453) was purchased from MedChem Express. Hydroxytyrosol (#S3826), 3,4-dihydroxyphenylacetic acid (#S5639), gallic acid ethyl ester (#S5550), epigallocatechin (#S3922), 3,4-dihydroxyphenyl propionic acid (#S6338), epigallocatechin gallate (#S2250) were purchased from Selleckchem (Houston, TX, United States of America). Gallocatechin (#G0243) was purchased from LKT Labs (St.Paul, MN, United States of America). Gallotannin and dietary polyphenols shown in Table 1 A were kindly provided by Dr. Hsun-Shuo Chang (School of Pharmacy, College of Pharmacy, Kaohsiung Medical University), Dr. Hui-Chi Huang (School of Chinese Medicine and Graduate Institute of Chinese Medicine, China Medical University), and Dr. Chung-Kuang Lu (National Research Institute of Chinese Medicine).

**TABLE 1:** Antagonistic potency against BTZ of polyphenols.

Full name	Abbreviation	Number of VDM^a^	MAC^b^ (μM)	Viability difference (%)	AI^c^
A. Gallotannin					
2-isopropyl-*O*‐β‐(6′‐*O*‐ galloyl)‐ glucopyranoside	IG	2	12.5	49.1	41
4-hydroxy-3-methoxyphenol 1-*O*-β-D-(2′,6′-di-*O*-galloyl) glucoside	2′,6′-di GG	4	1.56	28.6	9
1,3,6-tri-*O*-galloyl-β-D-glucose	1,3,6-triGG	6	0.78	53.3	2
1,2,6- tri-*O*-galloyl-β-D-glucose	1,2,6-triGG	6	1.56	99.3	3
1,4,6-tri-*O*-galloyl-β-D-glucose	1,4,6-triGG	6	1.56	43.8	6
corilagin	Cori-triGG	6	3.13	52.2	10
1,2,3-tri-*O*-galloyl-beta-D-glucose	1,2,3-triGG	6	12.5	47.6	42
1,2,3,6-tetragalloyl-beta-D-glucose	1,2,3,6-tetraGG	8	1.56	76.2	3
1,2,3,4,6-penta-*O*-galloyl-β-D-glucose	PGG	10	0.78	98.5	1
tellimagrandin II	Telli-PGG	10	0.78	35.8	3
B. Dietary polyphenols					
protocatechuic acid	PA	1	>50	-	>300
3,4-dihydroxyphenylpropionic acid	DPA	1	>50	-	>300
caffeic acid	CA	1	>50	-	>300
(+)‐catechin	CC	1	>50	-	>300
(−)‐epicatechin	EC	1	>50	-	>300
hydroxytyrosol	HT	1	>50	-	>300
3,4‐dihydroxyphenylacetic acid	DAA	1	50	64.8	124
quercetin	QC	1	3.13	35.9	14
luteolin	LT	1	12.5	25.6	78
gallic acid	GA	2	12.5	64.3	31
gallic acid ethyl ester	GE	2	25	47.4	84
(−)‐gallocatechin	GC	2	6.25	41.3	24
(−)‐epigallocatechin	EGC	2	12.5	108.2	18
(−)-epicatechin-3-gallate	ECG	3	25	69.1	58
epigallocatechin gallate	EGCG	4	3.13	91.5	5
7,8,3′,4′-tetrahydroxyflavone^c^	tetra-FV	2	10	∼59	∼17
fisetin^d^	FS	1	20	∼59	∼34
myricetin^d^	MC	2	∼10	∼39	∼26
rutin hydrate^d^	RH	1	>100	-	>345
vitamin C^e^	Vitamin C	1	125	∼59	∼212

^a^ VDM: vicinal diol moieties.

^b^ MAC (Minimal antagonistic concentration): the minimal concentration of polyphenols (in nM) required to counteract more than 25% of the viability reduction caused by BTZ.

^c^ Antagonistic index (AI) = MAC, of polyphenol/[(Concentration of BTZ)x(Viability Difference)]. Concentration of BTZ, is the amount (in nM) of BTZ, used in the viability experiment. Viability Difference represents the extent to which polyphenols reverse BTZ’s cytotoxic effects at their MAC, concentration. It is calculated as ViaPP/BTZ, ViaBTZ, where Via represents cell viability (in %). ViaPP/BTZ, is the viability when both polyphenol and BTZ, are applied together, while ViaBTZ, is the viability with BTZ, alone.

^d^ MAC, was adopted from *Br J Haematol. 2009, 146: 270*.

^e^ MAC, of vitamin C was adopted from *Leukemia. 2009, 23:1679*.

### 2.2 Cell culture

Human MM cell lines (RPMI8226, NCI-H929, MM1S cell lines) were cultured as described in a previous paper (Tseeleesuren et al., 2018b). In brief, MM cells were cultured in RPMI1640 media supplemented with 10% fetal bovine serum (FBS), L-glutamine (2 mM), HEPES (10 mM), sodium pyruvate (1 mM), and 1% penicillin/streptomycin (100 unit/mL penicillin and 100 μg/mL streptomycin). MM cells were incubated at 37°C in a humidified incubator containing 5% CO2 in the air.

### 2.3 Cell viability assay and antagonistic potency evaluation

Cell viability assays were performed based on methods described in a previous paper (Tseeleesuren et al., 2018b). To compare the antagonistic potency of polyphenols among different studies that use varying BTZ concentrations and to more accurately represent the antagonistic potency of polyphenols, we introduced the antagonistic index (AI). AI values were calculated based on the polyphenol minimal antagonistic concentration (MAC), BTZ concentration, and their impact on cell viability. The AI is computed as:

$$\text{AI}=\frac{\text{MAC}\;\text{of}\;\text{polyphenol}}{\left(\text{Concentration}\;\text{of}\;\text{BTZ}\right)\times \left(\text{Viability}\;\text{Difference}\;\right)}$$

Definitions:

• MAC: The minimal concentration of polyphenols (in nM) required to counteract more than 25% of the viability reduction caused by BTZ.
• Concentration of BTZ: Amount (in nM) of BTZ used in the viability experiment.
• Viability Difference represents the extent to which polyphenols reverse BTZ’s cytotoxic effects at their MAC concentration. It is calculated as ViaPP/BTZ - ViaBTZ, where Via represents cell viability (in %). ViaPP/BTZ is the viability when both polyphenol and BTZ are applied together, while ViaBTZ is the viability with BTZ alone.

A lower AI value signifies a polyphenol with a higher antagonistic potency against BTZ.

### 2.4 ChemGPS-NP analysis

The SMILES notations of polyphenols were obtained using ChemDraw 18.1. These SMILES notations were then input into the online tool ChemGPS-NPWeb (http://chemgps.bmc.uu.se) to obtain the ChemGPS-NP scores, as described previously (Larsson et al., 2007; Rosén et al., 2009; Korinek et al., 2017). From ChemGPS-NPWeb, eight principal components (PCs) derived from 35 chemical descriptors were obtained, serving as a tool for navigating biologically relevant chemical space (Supplementary Table S1). For this study, we primarily used the first three principal components (PC1, PC2, and PC3), which account for 71% of the variance in the data. PC1 represents size, shape, and polarizability; PC2 represents aromatic and conjugation-related properties; and PC3 represents lipophilicity, polarity, and hydrogen bond capacity. The values of PC1, PC2, and PC3 for all polyphenols were then plotted using Grapher 2.5 (Mac OS) to generate three-dimensional (3D) plots depicting the ChemGPS-NP chemical property space.

### 2.5 Long-term cytotoxicity treatment

The long-term BTZ treatment protocol was based on the dosing schedule used for MM patients. In brief, RPMI8226 cells were seeded at a density of 2.5 × 10^4^/mL. These cells were then treated with BTZ at a concentration of 3.125 nM in the presence or absence of GA at 1 μM, EGC at 0.73 µM, or EGCG at 0.17 µM on days 1, 4, 8, and 11. Cell images were captured using a bright-field microscope. Cell counts were determined using the trypan blue exclusion method on days 8, 11, and the final day of the experiment.

### 2.6 Proteasome activity assay

Proteasome activity assays were conducted based on a previous paper (Maher, 2014), with some modifications. RPMI8226 cells (10^5^ cells/10 mL) treated with BTZ or polyphenols following the above-described long-term BTZ treatment protocol were harvested on day 8, lysed with 0.5% NP-40, and 50 µg protein was subjected to proteasome activity assays using Suc-LLVY-AMC substrate (P802, Enzo Life Sciences). Fluorescence output was detected using a Synergy HT Multimode Microplate Reader (Biotek Instruments, Winooski, VT, UnitedStates).

### 2.7 Combination index

To evaluate the potential synergistic antagonism effects of 1,3,6-triGG and PGG against BTZ, we first calculated the viability difference between the cell viability of 1,3,6-triGG and PGG, both alone and in combination, in the presence of BTZ, and the cell viability when BTZ was applied alone. Subsequently, we inputted the viability differences caused by 1,3,6-triGG and PGG, both alone and in combination, into CompuSyn software version 1.0. Finally, we generated the combination index (CI) plot using the Chou-Talalay method (Chou et al., 1994).

### 2.8 Statistical analysis

Statistical analysis was conducted with GraphPad Prism 8.0, using one-way and two-way ANOVA to assess group differences. Results are shown as mean ± SD, with *p* < 0.05 indicating significance.

## 3 Results

### 3.1 Vicinal diol moiety counts and configuration influence galloyl glucose gallotannins’ antagonism against BTZ

**FIGURE 1:**
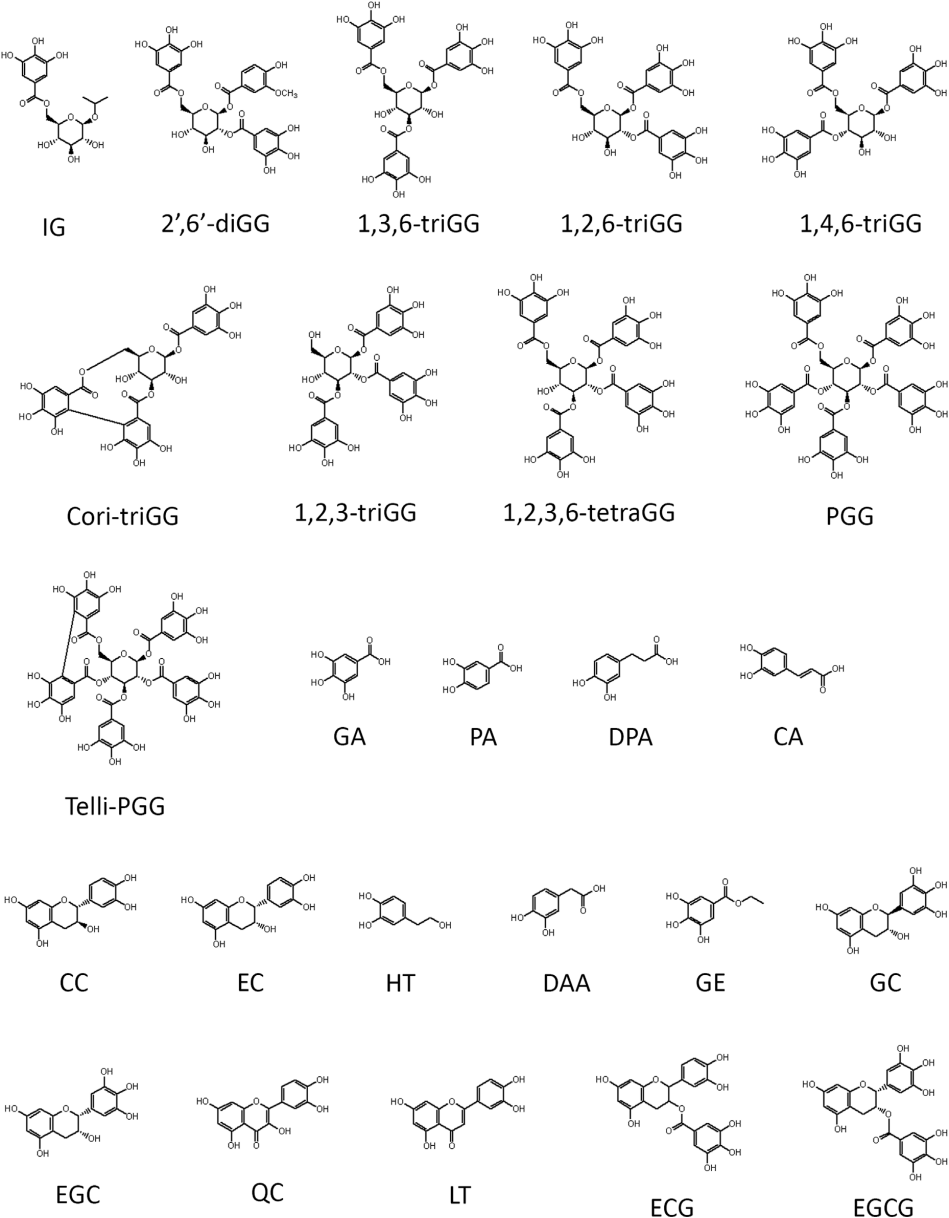
Structure summary of polyphenols utilized in this study. Ten gallotannins used in this study include 2-isopropyl-*O*‐β‐(6′‐*O*‐galloyl)‐glucopyranoside (IG), 4-hydroxy-3-methoxyphenol 1-*O*-β-D-(2′,6′-di-*O*-galloyl) glucoside (2′,6′-diGG), 1,3,6-tri-*O*-galloyl-β-D-glucose (1,3,6-triGG), 1,2,6-tri-*O*-galloyl-β-D-glucose (1,2,6-triGG), 1,4,6-tri-*O*-galloyl-β-D-glucose (1,4,6-triGG), corilagin (Cori-triGG), 1,2,3-tri-*O*-galloyl-beta-D-glucose (1,2,3-triGG), 1,2,3,6-tetragalloyl-beta-D-glucose (1,2,3,6-tetraGG), 1,2,3,4,6-penta-*O*-galloyl-β-D-glucose (PGG), tellimagrandin II (Telli-PGG). Fifteen dietary polyphenol used in this study consist of gallic acid (GA), protocatechuic acid (PA), 3,4-dihydroxyphenylpropionic acid (DPA), caffeic acid (CA), (+)‐catechin (CC), (−)‐epicatechin (EC), hydroxytyrosol (HT), 3,4-dihydroxyphenylacetic acid (DAA), gallic acid ethyl ester (GE), (−)‐gallocatechin (GC), (−)‐epigallocatechin (EGC), quercetin (QC), luteolin (LT), (−)-epicatechin-3-gallate (ECG), and epigallocatechin gallate (EGCG).

**FIGURE 2:**
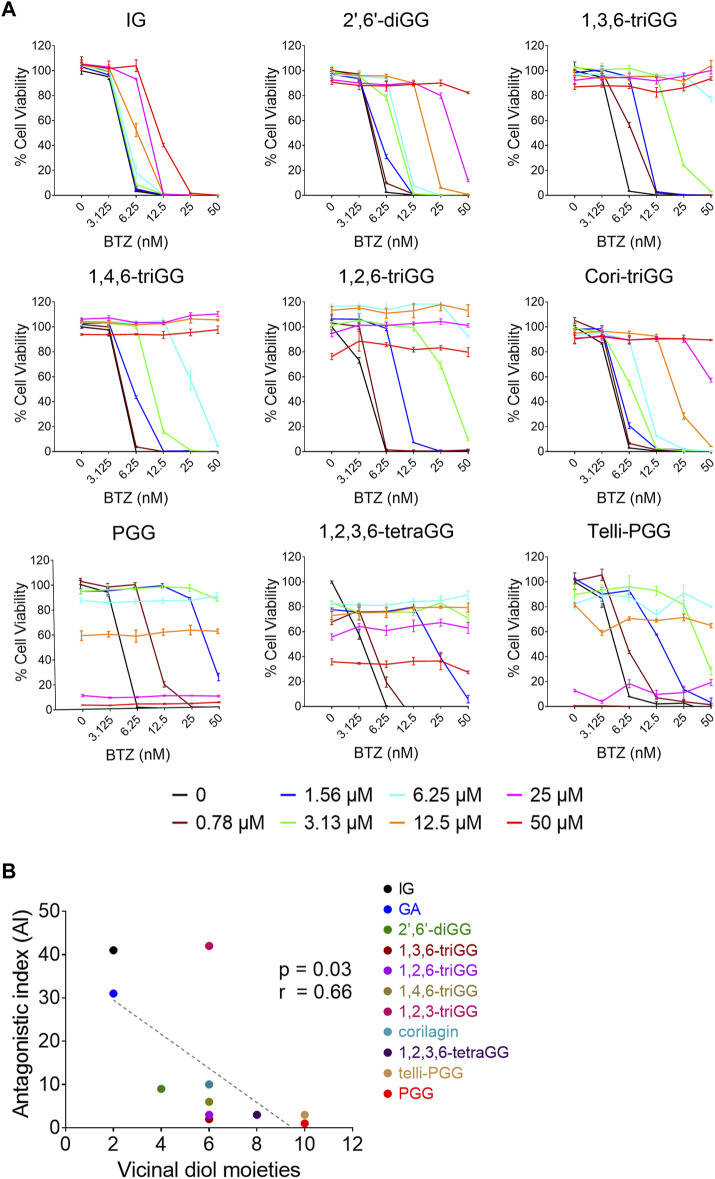
The antagonistic effects against BTZ of gallotannins are positively correlated with their number of vicinal diol moieties (VDMs). **(A)** RPMI 8226 cells were treated with polyphenols and BTZ for 72 h, then cell viability and AI were measured. Data: mean ± SD (n = 3). **(B)** Correlation between number of VDMs in 11 polyphenols and their AI values. The antagonistic effects of GA and 1,2,3-triGG toward BTZ were showed in Supplementary Figure S1. Each data point represents one polyphenol, with linear regression analysis shown in a dotted line.

Our previous study demonstrated that 1,2,3,4,6-penta-O-galloyl-β-D-glucose (PGG), a galloyl glucose gallotannin, which is a type of polyphenol, with a central glucose core esterified to five gallic acid units, which yields 10 vicinal diol moieties (VDMs) (Figure 1), exhibits a potent inhibitory effect against BTZ (Tseeleesuren et al., 2018a). To verify the relationship between the number of VDM in polyphenols and their counteractive effects against bortezomib (BTZ), we conducted cell viability assays using combinations of various BTZ concentrations and 10 galloyl glucose gallotannin, each with differing VDM counts. In addition to PGG, we examined 9 other galloyl glucose gallotannins. These include 2-isopropyl-*O*-‐β‐(6′‐O‐galloyl)‐glucopyranoside (IG) with 2 VDMs; 4-hydroxy-3-methoxyphenol-1-O-β-D-(2′,6′-di-O-galloyl)-glucoside (2′,6′-diGG) with 4 VDMs; 1,3,6-tri-O-galloyl-β-D-glucose (1,3,6-triGG), 1,2,6-tri-O-galloyl-β-D-glucose (1,2,6-triGG), 1,4,6-tri-O-galloyl-β-D-glucose (1,4,6-triGG), corilagin (Cori-triGG), and 1,2,3-tri-O-galloyl-beta-D-glucose (1,2,3-triGG), all with 6 VDMs each; 1,2,3,6-tetragalloyl-beta-D-glucose (1,2,3,6-tetraGG) with 8 VDMs; and Tellimagrandin II (telli-PGG) with 10 VDMs (Figure 1). Additionally, we included the monomer of gallotannins, gallic acid (GA), which possesses 2 VDMs. At 6.25 nM BTZ, these polyphenols significantly enhanced cell viability within 72 h, diminishing BTZ´s anticancer effects (Figure 2A; Supplementary Figure S1). To assess the antagonistic potency, we computed the antagonistic index (AI) for each polyphenol. We observed that IG exhibited comparable antagonistic effects with GA (Table 1). AI reflected that higher VDM counts led to stronger antagonistic actions against BTZ, with AIs ranging from 1 to 6 for galloyl glucoses with more than 6 VDMs, and exceeding 10 for those with fewer than 4 VDMs (Table 1; Figure 2B). Interestingly, 1,2,3-triGG, despite having 6 VDMs, displayed reduced antagonistic potency (AI = 42) compared to other 6-VDM compounds (AIs ranging from 2 to 10) (Table 1; Figure 2A; Supplementary Figure S1). Thus, specific positioning of the galloyl group on the carbon-6 of the glucose core significantly enhanced antagonistic activity. Furthermore, crosslinking galloyl groups in cori-triGG and telli-PGG impaired their antagonistic potency, as compared to 1,3,6-triGG and PGG, respectively. These findings highlight that while VDM count primarily influences the antagonistic effect against BTZ-induced cell death, structural features such as the position and flexibility of the galloyl group significantly contribute to this antagonistic effect.

### 3.2 Origin of the VDMs but not total VDM concentration play crucial role in the combinatory antagonistic effects against BTZ

**FIGURE 3:**
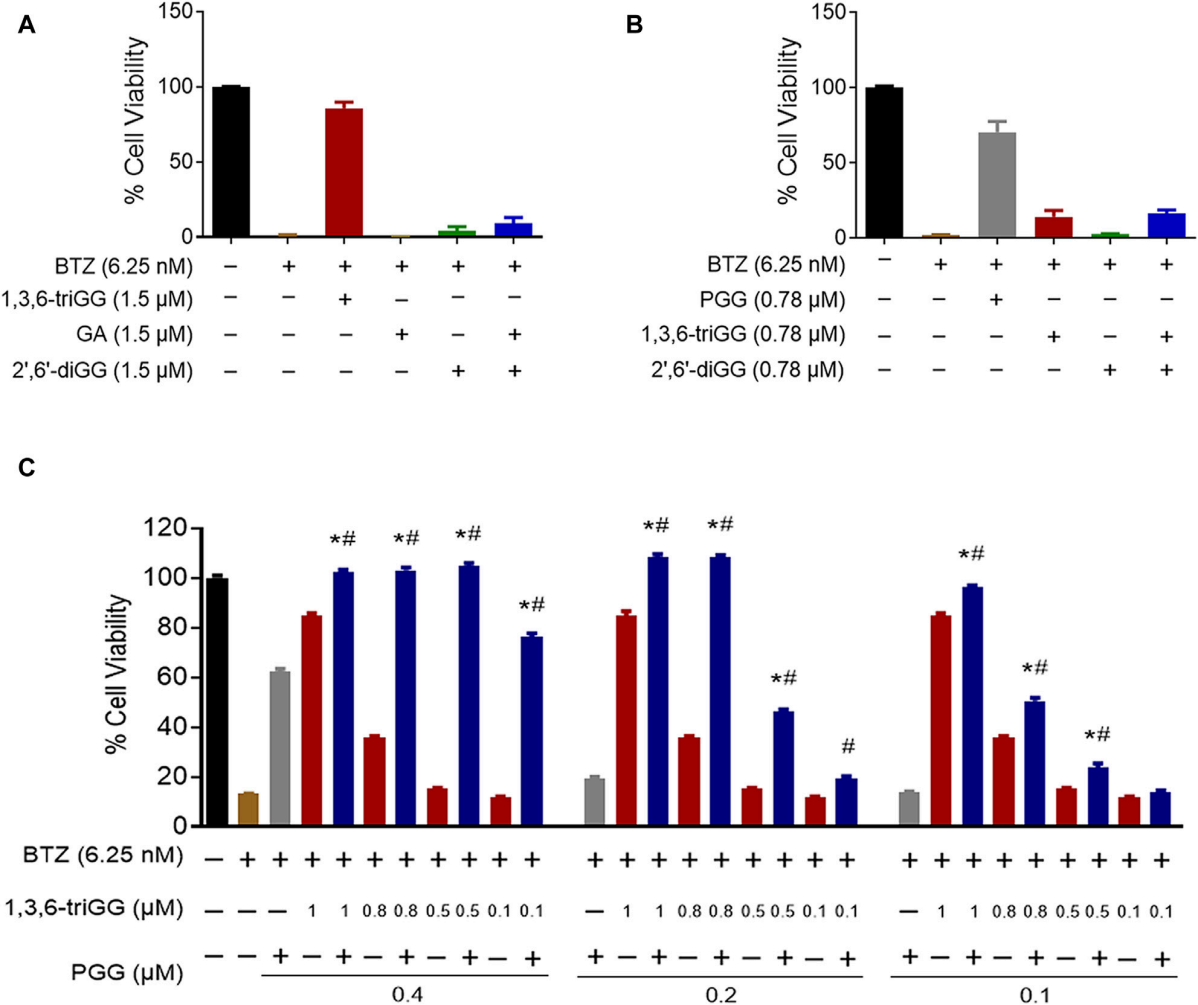
Origin of the VDMs but not total VDM concentration play crucial role in the combinatory antagonistic effects against BTZ in gallotannins. RPMI 8226 cells were treated with the indicated concentrations of BTZ and polyphenols both alone and in combination for 72 h, then cell viability was measured by resazurin. **(A)** The antagonistic effects of 1,3,6-triGG and the combination of GA and 2′,6′-diGG against BTZ. **(B)** The antagonistic effects of PGG and the combination of 1,3,6-triGG and 2′,6′-diGG against BTZ. **(C)** The antagonistic effects of 1,3,6-triGG and PGG against BTZ. Data are presented as mean ± SD (n = 3). The (*) and (#) indicate a significant difference compared with the corresponding PGG alone group and 1,3,6-triGG alone group, respectively (*p* < 0.05, one-way ANOVA).

We investigated if equivalent concentrations of VDM consistently yield similar antagonistic effects, comparing 1,3,6-triGG (6 VDMs) to a combination of GA (2 VDMs) and 2′,6′-diGG (4 VDMs). At 1.5 μM, 1,3,6-triGG significantly inhibited BTZ-induced cytotoxicity (6.25 nM), while the combination of 1.5 μM GA and 1.5 μM 2′,6′-diGG did not show comparable effects (Figure 3A). Similar patterns were observed with PGG (10 VDMs) at 0.78 μM compared to a combination of 2′,6′-diGG (4 VDMs) and 1,3,6-triGG (6 VDMs) at 0.78 μM (Figure 3B). However, combining 1,3,6-triGG and PGG demonstrated synergistic antagonistic effects against BTZ-induced cytotoxicity (Figure 3C; Table 2). These findings suggest that the antagonistic capacity is not solely determined by the total VDM concentration, but the origin of the VDMs play a crucial role.

**TABLE 2:** Combination index (CI) of 1,3,6-triGG and PGG.

1,3,6-triGG (µM)	PGG (µM)	CI value
1.1	0.39	0.93
	0.19	0.51
	0.098	0.75
0.78	0.39	0.76
	0.19	0.4
	0.098	1.3
0.5	0.39	0.59
	0.19	1.3
	0.098	1.76
0.1	0.39	0.86
	0.19	1.61
	0.098	2.02

CI, was obtained using Compusyn 1.0 software.

### 3.3 The aromaticity of dietary polyphenols contributed to their antagonistic effect against BTZ

**FIGURE 4:**
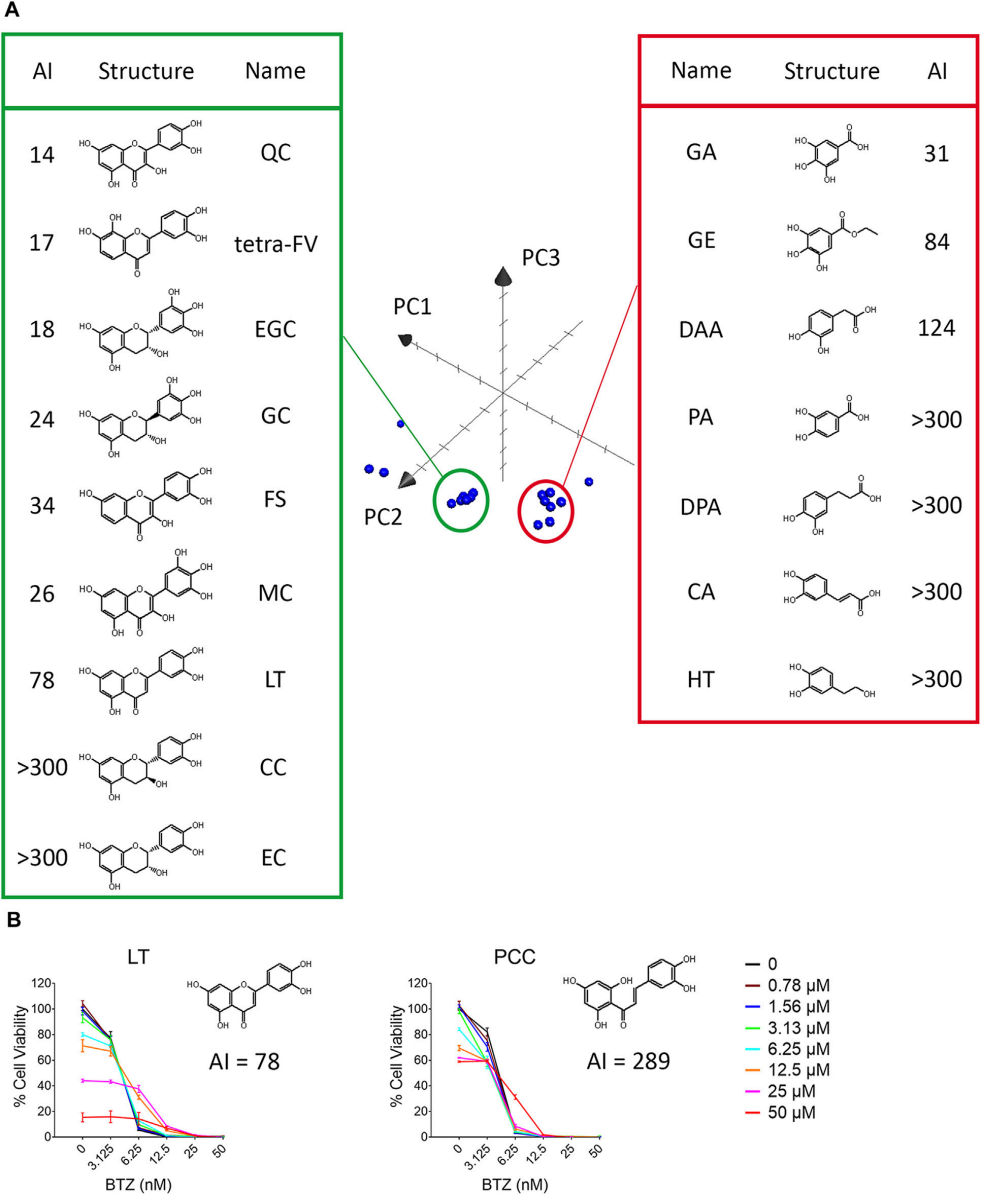
The aromaticity of dietary polyphenols contributed to their antagonistic effect against BTZ. **(A)** ChemGPS-NP analysis of the structure-activity relationship of vicinal diol-containing dietary polyphenols. Twenty dietary polyphenols, including 15 dietary polyphenols, 4 analogs of quercetin and vitamin C (Table 1) were plotted into the three-dimensional graph consisting of PC1, PC2, PC3 in Apple™ system software Grapher 2.0. **(B)** The antagonistic potency against BTZ of LT and 2′,4′,6′,3,4-pentahydroxychalcone (PCC) was detected after 72 h by resazurin in RPMI 8226 cells. The data represent the mean ± SD (n = 3).

Next, we examined the antagonistic potency of 15 dietary polyphenols (Figure 1), chosen for their frequent detection in human blood and possession of VDM (Achaintre et al., 2018). These include GA, protocatechuic acid (PA), 3,4-dihydroxyphenylpropionic acid (DPA), caffeic acid (CA), (+)-catechin (CC), (−)‐epicatechin (EC), hydroxytyrosol (HT), 3,4-dihydroxyphenylacetic acid (DAA), gallic acid ethyl ester (GE), (−)‐gallocatechin (GC), (−)‐epigallocatechin (EGC), quercetin (QC), luteolin (LT), (−)-epicatechin-3-gallate (ECG), and epigallocatechin gallate (EGCG). Contrary to expectations, the number of VDMs did not directly correlate with their antagonistic potency (Table 1; Supplementary Figures S2, S3). For example, ECG (3 VDMs) had an AI of 58, while QC, with only 1 VDM, showed an AI of 14. Additionally, compounds with the same VDM count, like DAA, LT, and QC, displayed AI values ranging from 14 to over 300 (Table 1). ChemGPS-NP analysis and 3D graphing revealed two clusters, suggesting that size (PC1) and aromaticity (PC2) play key roles in the antagonistic effects of dietary polyphenols (Figure 4A). Despite structural similarities, there´s a significant (∼5-fold) difference in antagonistic activity between QC (AI = 14) and LT (AI = 78), emphasizing the importance of the hydroxyl group at C-3 in the C-ring, present in QC but absent in LT. Evaluations of QC, CC, and EC, all possessing a C3-OH group, revealed weak antagonistic effects for CC and EC due to their non-aromatic C-ring. Comparing LT with 2′,4′,6′,3,4-pentahydroxychalcone (PCC), which maintains double bond conjugation, showed PCC´s very weak antagonistic effect (AI = 298) (Figure 4B). In conclusion, while C3-OH plays a definitive role, aromaticity primarily dictates flavonoids’ antagonistic effects against BTZ.

### 3.4 Common dietary polyphenols at physiological concentrations synergistically counteract BTZ effects

**FIGURE 5:**
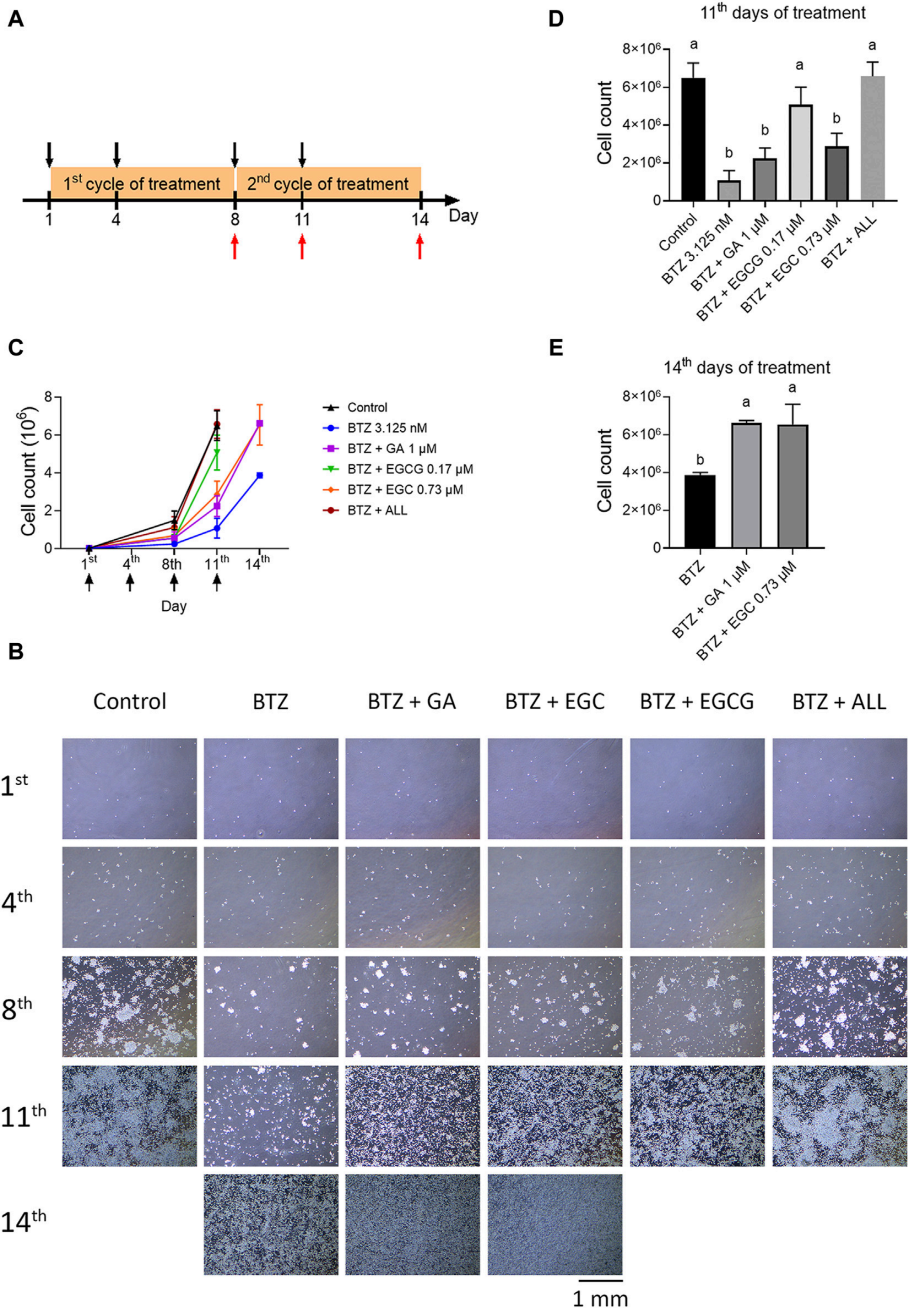
The combination of GA, EGC, and EGCG at physiological concentrations completely reverse the effect of BTZ. **(A)** Treatment Protocol: RPMI 8226 cells were cultured in 10 cm dishes (2.5 × 10^4^ cells/dish) and treated with BTZ (3.125 nM), along with gallic acid (GA, 1 µM), (−)‐epigallocatechin (EGC, 0.73 µM), and epigallocatechin gallate (EGCG, 0.17 µM), bringing the total volume to 10 mL, for a period of 3 days. On day 4, an additional 5 mL of the drug-containing medium was added to the dishes, followed by a continued incubation for 4 days. On day 8, the medium was refreshed with 10 mL of the drug-containing medium. Subsequently, on day 11, another 5 mL of the drug-containing medium was introduced and incubation was continued to the end of experiment. Cell counts were conducted on days 8, 11, and 14. Treatment days and cell count days are indicated by black and red arrows, respectively. **(B)** Phase-contrast microscopic images of cell density on the 1st, 4th, 8th, 11th, and 14th day. Pictures were taken at ×4 magnification. **(C)** Growth curve of all groups. The cell numbers were determined using trypan blue. The treatment day was indicated by the black arrow. **(D,E)** Total cell counts on the 11th and 14th day, with statistical analysis. Data represent the mean ± SD (n = 3). Statistical significance: *p* < 0.05 (one-way ANOVA, Tukey’s multiple comparisons test).

To investigate the potential antagonistic effects of common dietary polyphenols against BTZ, we selected GA, EGC, and EGCG based on their abundance in foods and relatively high level in human plasma. The experiment´s design is depicted in Figure 5A. RPMI8226 cells were exposed to BTZ at 3.125 nM, approximating the average blood concentration (ABC), and to selected polyphenols: GA (1 µM), EGC (0.73 µM), and EGCG (0.17 µM), either individually or in combinations. Long-term treatment revealed that repeatedly treating cells with BTZ at its ABC induced marked cytotoxicity by the 11th day. By this same time frame, EGCG alone was already displaying noticeable antagonistic effects against BTZ (Figures 5B–D). By day 14, GA and EGC individually mitigated BTZ-induced cell growth reduction (Figures 5B, C, E). Notably, the combined treatment with GA, EGC, and EGCG showed substantial antagonistic effects by day 11 (Figures 5B–D), and restoring proteasome activity by day 8 (Supplementary Figure S4). These findings highlight a synergistic antagonism against BTZ of GA, EGC, and EGCG at physiologically attainable concentrations.

## 4 Discussion

MM is a hematological malignancy characterized by the uncontrolled clonal proliferation of plasma cells derived from B cells within the bone marrow cavity. BTZ is an injectable anti-cancer agent that is utilized in the treatment of MM at various stages of the disease. Although BTZ has demonstrated significant clinical efficacy in the treatment of MM, the development of resistance to BTZ is common. Various mechanisms have been identified in relation to the resistance and relapse of MM patients treated with BTZ (Gonzalez-Santamarta et al., 2020). In this study, we explore an additional possibility: the antagonistic effects against BTZ of polyphenols, a vast group of natural products commonly found in food and beverages. The vicinal diol group of polyphenols can react with the boronic group of BTZ and neutralize BTZ´s anticancer effect (Golden et al., 2009; Kim et al., 2009; Jia and Liu, 2013; Tseeleesuren et al., 2018b). Our findings indicate that the number of vicinal diol moieties, their spatial arrangement, and the aromaticity of polyphenols are critical factors determining their antagonistic effects. Moreover, we demonstrated that three common dietary polyphenols—GA, EGC, and EGCG—effectively counteract BTZ´s anticancer effects, both individually and in combination in long-term treatment experiments. Most importantly, the combination of these dietary polyphenols, even at physiologically achievable concentrations, showed very potent antagonistic effects, nearly completely abolishing BTZ´s cytotoxic effect. Thus, it is reasonable to speculate that uptake of particular polyphenols could mitigate the anticancer effects of BTZ in MM patients and contribute to resistance and relapse of MM.

Several earlier publications have demonstrated that the antagonistic effects of polyphenols on BTZ were observed in MM cells derived from 6 MM patients, as well as in several MM cell lines such as MM1, NCI-H929, U266, and MC/CAR cells (Liu et al., 2008; Golden et al., 2009; Kim et al., 2009; Tseeleesuren et al., 2018b). Unlike RPMI-8226, in both MM1S and NCI-H929 cells, BTZ at 3 nM caused approximately 80% cytotoxicity. Not surprisingly, EGCG, even at concentrations below 1.56 μM, increased cell viability by approximately 25% in both MM1S and NCI-H929 cells (Supplementary Figure S5). Furthermore, the antagonistic index (AI) values for MM1S and NCI-H929 are 18 and 10, respectively, which are slightly higher than in RPMI-8226 cells (AI = 5) [Note: the AI of polyphenols without antagonistic activity are higher than 300]. These findings indicate that sensitivity to BTZ could slightly affect the antagonistic activities of polyphenols against BTZ. Considering the variety of cell lines representing tumor heterogeneity, it can be reasonably inferred that even at physiological concentration ranges, polyphenols can neutralize a portion of BTZ. The remaining BTZ could still be sufficient to eliminate more sensitive populations of MM cells; however, the amount may not be enough for less sensitive populations, thus resulting in cancer relapse.

According to our observation, the antagonistic potency of polyphenols against BTZ is influenced by two factors: the concentration of BTZ used to cause cytotoxicity and the extent to which polyphenols can reduce BTZ-caused cytotoxicity. Previous studies have used quite high concentrations of BTZ (equal to or higher than 10 nM) to investigate the antagonistic effects of polyphenols (Liu et al., 2008; Golden et al., 2009; Kim et al., 2009). The mechanism behind these effects involves the interaction between the vicinal diol moieties of polyphenols and the boronic acid of BTZ, necessitating higher concentrations of polyphenols to counteract higher concentrations of BTZ. Therefore, MAC cannot be used to compare our results (6.25 nM and lower concentration of BTZ were used in our study) with data from previous papers. Furthermore, we observed that two polyphenols with the same MAC can exhibit quite different antagonistic potencies. For example, both 1,2,6-triGG and 1,4,6-triGG have a MAC of 1.56 μM. However, 1,2,6-triGG (AI = 3) can decrease BTZ toxicity by 99.3%, while 1,4,6-triGG (AI = 6) can only decrease it by 43.8% (Table 1). Therefore, to compare the antagonistic potency of polyphenols among different studies (using different BTZ concentrations) and to more accurately represent the antagonistic potency of polyphenols, we introduced the antagonistic index (AI) in this manuscript for the first time. The AI takes into account MAC, the concentration of BTZ, and the reversing levels. We believe that the AI value can more concisely indicate which polyphenols are potent in neutralizing BTZ’s anti-MM effect.

It is noteworthy that almost all gallotannins displayed a strong antagonistic potency against BTZ. Potent gallotannins could further exhibit a synergistic effect against BTZ. Moreover, other dietary polyphenols, such as QC, also demonstrated potent antagonistic effects against BTZ. However, we opted not to use these compounds for extended treatment experiments, given that these polyphenols are known to have low bioavailability or could undergo extensive metabolism in the body (Luca et al., 2020; Alizadeh and Ebrahimzadeh, 2022). Gallotannins are commonly perceived to be poorly absorbed due to their high molecular weight and polarity (Marcińczyk et al., 2021; R et al., 2021). QC primarily exists in its original structure within the gastrointestinal (GI) tract and is metabolized by the small intestine and liver before entering the circulatory system. As a result, it circulates in the blood mainly in conjugated forms, which might lose their vicinal diol moieties, diminishing the antagonistic activity (Almeida et al., 2018). However, does this mean that these potent BTZ antagonists can be overlooked when MM patients receive boronic acid–based PI treatments? Interestingly, the first oral proteasome inhibitor, Ixazomib (IXZ), has been frequently used to treat MM and even solid tumors (Raedler, 2016; Harris et al., 2020). IXZ is formulated as a citrate ester of boronic acid (IXZ citrate) and rapidly hydrolyzes to its biologically active form when exposed to aqueous solutions in the GI tract and plasma (Muz et al., 2016). This prompts the question: will these poorly absorbed yet potent BTZ antagonists, such as QC and gallotannins, effectively block Ixazomib (IXZ) in the GI tract? As anticipated, our findings indicate that PGG strongly counteracts the cytotoxic effect of the active form of IXZ (Supplementary Figure S6). Since it is known that PGG and EGCG do not have antagonistic activity against non-boronic acid–based PIs (Modernelli et al., 2015; Tseeleesuren et al., 2018b), our research collectively suggests that patients undergoing treatment with IXZ or BTZ should be cautious about their intake of foods rich in gallotannins or polyphenols.

A vicinal diol is a chemical group with two hydroxyl groups on neighboring carbon atoms in a molecule. This raises a question about the number of VDMs in gallic acid (GA), which contains a pyrogallol unit. Pyrogallol, or 1,2,3-trihydroxybenzene, has three hydroxyl groups on adjacent carbon atoms in its benzene ring. Pyrogallol can be considered to have two VDMs, but the central hydroxyl group is shared. Notably, when one VDM reacts with a boronic group using its two hydroxyls, no VDM remains, leaving only one hydroxyl on pyrogallol. Interestingly, our findings show that GA (3,4,5-trihydroxybenzoic acid, AI = 31) is about ten times more potent than protocatechuic acid (PA or 3,4-dihydroxybenzoic acid, AI>300), which has a catechol unit (1 VDM) with two adjacent hydroxyls on its benzene ring. This implies that despite the shared hydroxyl group in the two VDMs of pyrogallol, it is more reactive to boronic acid than catechol. Therefore, we used VDM to estimate potential reactive moieties and considered the pyrogallol group as two VDMs in our calculations.

Flavonoids, comprising one of the most extensive classes of polyphenols, encompass over 8,000 distinct compounds identified across a broad spectrum of vascular plants. In our investigation of the counteractive potential of polyphenols against BTZ, we specifically focused on those detectable in human plasma and containing VDMs (Achaintre et al., 2018). Consequently, this study delved into assessing the antagonistic impact against BTZ within the flavan-3-ols, flavonols, and flavones subgroups. Our findings underscored the pivotal role of aromaticity and conjugation properties in the antagonistic effects exhibited by flavonoids. Additionally, we observed that the hydroxyl group at C-3 of the C-ring significantly influences the antagonistic effects against BTZ. For flavonoids with a single VDM in ring B, the descending order of antagonistic potency is as follows: flavonols (QC and FS) exhibit the highest potency, followed by flavone (LT), and then flavan-3-ols (CC and EC) with comparatively lower potency. Notably, this order of antagonistic potency varies in flavonoids with 2 VDMs, with flavone (tetra-FV) displaying the most potent effects, followed by flavan-3-ols (EGC and GC), and flavonols (MC). This observed difference may be attributed to the distinct positioning of VDMs. The two VDMs of tetra-FV are positioned completely separately, with one in ring A and the other in ring B. This configuration affords the opportunity for both of its VDMs to interact independently with two distinct BTZ molecules. On the contrary, the two VDMs of MC originate from its pyrogallol unit (B ring). As mentioned above when one VDM of pyrogallol engages with BTZ, no VDM remains. Hence, the number and arrangement of VDMs contribute to the antagonistic potency against BTZ. In addition, the AI of tetra-FV and MC were calculated according to cytotoxicity results referred from previous paper (Kim et al., 2009). This could account for the observed variation in the order of antagonistic potency among flavonoids. Drawing from the distinctive features of potent antagonists, it is plausible that flavonoids not included in this study, such as flavonols (azaleatin, gossypetin, rhamnetin), flavone (baicalein, negletein, norwogonin, 6-hydroxyluteolin, scutellarein, sorbifolin, isoscutellarein, tricetin, nepetin, pedalitin, nodifloretin, cirsiliol, hypolaetin, onopordin), and flavan-3-ols (fisetinidol, mesquitol, robinetinidol), may harbor antagonistic effects against BTZ. Nevertheless, the likelihood of these substances counteracting the anticancer effects of BTZ/IXZ through daily dietary intake is minimal due to their limited abundance in regular dietary sources (Jun et al., 2016; Murphy et al., 2019). However, as they may be active or major components in certain herbs, caution is advised when consuming herbal products containing these flavonoids.

Among all dietary polyphenols, we are particularly interested in the antagonistic potential of GA, EGCG, and EGC at their physiological concentrations, due to their ubiquity and potential to achieve higher plasma concentrations. GA is a naturally occurring polyphenol found in a variety of fruits, vegetables, teas, and herbal medicines, commonly present in the foods we consume daily. The average adult intake of GA is estimated to be around 25 mg/day, based on studies of dietary consumption (Grosso et al., 2014). Pharmacokinetic studies have shown that an intake of 25 mg of GA, whether as a pure compound or in tea, leads to plasma concentrations of approximately 0.9–1.1 μM (Manach et al., 2005). EGCG and EGC, which are prevalent in green tea, have also been examined. The daily consumption of tea is estimated at about 2.5 cups, with an average of 2.5 g of tea leaves per cup (Shen and Chen, 2008; Inoue-Choi et al., 2010; Additives et al., 2018). Pharmacokinetic studies suggest that after drinking approximately two cups of tea, plasma concentrations of EGCG and EGC can reach about 0.17 μM (77.9 ± 22.2 ng/mL) and 0.73 μM (223.4 ± 35.2 ng/mL), respectively (Lee et al., 2002). Although conventional cytotoxicity assays have not detected any antagonistic effects for these three polyphenols at physiologically achievable concentrations (as shown in Supplementary Figures S1, S2), our results indicate that a smaller quantity of polyphenols is sufficient to neutralize BTZ at lower concentrations. For instance, Figure 2 demonstrates that 1.56 μM PGG is needed to counteract 12.5 nM BTZ, and only 0.78 μM PGG is required for 6.25 nM BTZ. This suggests that polyphenol concentrations within the physiological range may be adequate to antagonize the anticancer effects of BTZ when its concentration is lower.

Regarding the blood concentration of BTZ, the recommended dosage and schedule is 1.3 mg/m2 on days 1, 4, 8, and 11 of a 21-day cycle, for up to eight cycles, administered intravenously (IV). The mean maximum plasma concentration reaches approximately 744 nM (286 ng/mL) post-IV administration. However, drug plasma concentrations typically decline rapidly to about 2.6 nM within 4 hours and remain mainly at this concentration until subsequent doses (with approximately 72 h between injections) (Philippe et al., 2008). Therefore, it is conceivable that dietary polyphenols at their physiological concentration might counteract BTZ at its average blood concentration. Nonetheless, at approximately 3 nM, BTZ does not exhibit noticeable toxicity in standard cytotoxicity assays. This could be a limitation of tetrazolium or resazurin-based cytotoxicity assays, where treatment duration is usually short (less than 3 days), often involving only a single dose, and therefore the effective concentrations might be higher than what is achievable in the human bloodstream. To investigate this further, we explored whether repeated treatments to maintain concentration over time are sufficient for BTZ to exhibit its anticancer effect at this level. We tested this hypothesis by repeatedly treating RPMI8226 cells with BTZ at 3.125 nM in a 10-cm dish on days 1, 4, 8, and 11. In the control group, an exponential growth curve was observed, with a cell count exceeding 6 million, approaching confluence on day 11. However, with repeated BTZ treatment at 3 nM, cells grew much slower, entering the exponential phase only after the final treatment. These findings suggest that our *in vitro* long-term treatment model can, to some extent, reflect the therapeutic effect of BTZ at its average blood concentration. Using this model, we demonstrated that GA, EGCG, and EGC at their physiological concentrations could counteract the anticancer effects of BTZ, and when treated in combination, they nearly completely abolished BTZ´s effects.

Based on our findings, individuals undergoing treatment with boronic acid-based proteasome inhibitors should avoid consuming GA and flavonoids, particularly EGCG and EGC. These polyphenols are abundantly found in various foods and beverages, with tea being a significant source of GA and flavonoids such as EGCG, EGC, QC, and MC (Manach et al., 2004; Murphy et al., 2019). In light of these discoveries, it is recommended to consider reducing the intake of polyphenol-rich foods (especially green tea) and polyphenol supplements for MM patients undergoing BTZ/IXZ treatment. Furthermore, future research should focus on examining the relationship between plasma concentrations of polyphenols in MM patients receiving BTZ/IXZ treatment and the influence of these concentrations on treatment outcomes.

Few studies have investigated the antagonistic effects of polyphenols on BTZ’s anti-MM activity. Golden et al. demonstrated the antagonistic capabilities of EGCG analogs against BTZ (Golden et al., 2009). Liu and colleagues explored the effects of QC and its analog on BTZ (Liu et al., 2008). Kim and team studied the antagonistic effects of 13 polyphenols, including one tannin, EGCG, GA, CA, RH, and eight flavonoids (Kim et al., 2009). In this study, we analyzed a series of 10 gallotannins and 15 dietary polyphenols that are detectable in human plasma. Our findings not only revealed structural features important for the antagonistic effects of polyphenols but also provide valuable insights that could significantly contribute to future research in this area.

## 5 Conclusion

Collectively, our findings indicated that the structural configuration, origin, and number of VDMs are crucial in determining the extent to which polyphenols counteract BTZ. Even at physiological levels, dietary polyphenols, especially from EGCG, EGC, and GA-rich foods like tea, may compromise BTZ´s anticancer efficacy. Hence, limiting polyphenol intake during BTZ treatment for MM is advisable.

## Data Availability

The original contributions presented in the study are included in the article/Supplementary Material, further inquiries can be directed to the corresponding authors.

## Abbreviations

1,2,3,6-tetraGG (1,2,3,6-tetragalloyl-beta-D-glucose); 1,2,3-triGG (1,2,3-tri-O-galloyl-beta-D-glucose); 1,2,6-triGG (1,2,6- tri-O-galloyl-β-D-glucose); 1,3,6-triGG (1,3,6-tri-O-galloyl-β-D-glucose); 1,4,6-triGG (1,4,6-tri-O-galloyl-β-D-glucose); 2′,6′-diGG (4-hydroxy-3-methoxyphenol 1-O-β-D-(2′,6′-di-O-galloyl) glucoside); ABC (average blood concentration; BTZ (bortezomib); CA (caffeic acid); CC (catechin); Cori-triGG (corilagin); DAA (3,4‐dihydroxyphenylacetic acid); DPA (3,4-dihydroxyphenylpropionic acid), EC (epicatechin); ECG (epicatechin-3-gallate); EGC (epigallocatechin); EGCG (epigallocatechin gallate); FS (fisetin); GA (gallic acid); GC (gallocatechin); GE (gallic acid ethyl ester); HT (hydroxytyrosol); IG (2- isopropyl O‐β‐(6′‐O‐ galloyl)‐ glucopyranoside); IXZ (Ixazomib); LT (luteolin); MAC (Minimal antagonistic concentration); MC (myricetin); MM (multiple myeloma); NVDM (the number of vicinal diol moieties); PA (protocatechuic acid); PGG (1,2,3,4,6-penta-O-galloyl-β-D-glucose); QC (quercetin); RH (rutin hydrate); Telli-PGG (tellimagrandin II); tetra-FV (7,8,3′,4′-tetrahydroxyflavone); VDMs (vicinal diol moieties).
