# Supplementary material for: The SAR analysis of dietary polyphenols and their antagonistic effects on bortezomib at physiological concentrations
Source: Front Pharmacol. 2024 Jul 25;15:1403424. doi: 10.3389/fphar.2024.1403424 (PMC11306019; doi:10.3389/fphar.2024.1403424)
Supplement: Supplementary file 1 [file Table1.pdf]

Supplement table 1. Principle component (PC) scores of 29 polyphenols and vitamin C from the ChemGPS-NP website

No.	Name	PC1	PC2	PC3	PC4	PC5	PC6	PC7	PC8
1	PGG	11.566	5.416364	-3.33789	-0.41292	-1.57953	-3.27749	-0.36899	-4.50216
2	1,3,6-triGG	5.932101	3.055839	-3.52528	-0.1976	-1.29181	-2.24785	-0.06913	-1.94221
3	1,2,6-triGG	5.932101	3.055839	-3.52528	-0.1976	-1.29181	-2.24785	-0.06913	-1.94221
4	1,2,3,6-tetraGG	8.75241	4.256728	-3.42914	-0.29072	-1.42959	-2.76925	-0.20519	-3.20777
5	Telli-pentaGG	11.39388	5.728904	-3.61565	-1.19136	-1.80254	-2.17115	-0.31648	-4.6615
6	4,3-di GG	5.508763	2.789414	-2.49851	0.007788	-1.18313	-2.11307	0.156138	-1.17208
7	1,4,6-triGG	5.932101	3.055839	-3.52528	-0.1976	-1.29181	-2.24785	-0.06913	-1.94221
8	Cori-triGG	5.746611	3.416041	-3.83604	-1.12155	-1.57203	-0.88329	-0.02199	-2.13592
9	QC	-0.63921	3.099599	-2.1874	-0.4549	-0.27888	0.038338	-0.10574	-0.86122
10	EGCG	2.771691	4.022871	-2.26123	-0.62083	-1.00634	-1.22342	-0.67976	-1.62133
11	tetraFV	-1.04905	3.264048	-1.59606	-0.39271	-0.12338	0.094525	-0.09987	-0.9856
12	GC	-0.52584	2.853466	-2.3318	-0.9622	-0.36608	-0.37347	-1.05196	-1.10101
13	EGC	-0.52584	2.853466	-2.3318	-0.9622	-0.36608	-0.37347	-1.05196	-1.10101
14	Fisetin	-1.0536	3.061221	-1.68306	-0.42472	-0.14354	0.149343	-0.02918	-0.44617
15	Myricetin	-0.22309	3.138994	-2.69222	-0.49005	-0.40717	-0.076	-0.18753	-1.27445
16	IG	0.162844	0.119748	-2.6121	-0.26161	-0.47123	-1.2843	-0.43552	-0.53397
17	1,2,3-triGG	5.932101	3.055839	-3.52528	-0.1976	-1.29181	-2.24785	-0.06913	-1.94221
18	GA	-2.9593	1.925473	-2.87773	0.175865	-0.27838	-0.29405	-0.24903	-0.57051
19	ECG	1.918386	4.040018	-1.75403	-0.67836	-0.41316	-1.02407	-0.94643	-1.91321
20	GE	-2.75799	1.483117	-1.83871	0.391609	0.057876	-1.05892	-0.29792	-0.75535
21	DAA	-3.1713	1.580864	-2.0245	0.215232	0.111091	-0.72847	-0.28042	-0.12754
22	LT	-1.04905	3.264048	-1.59606	-0.39271	-0.12338	0.094525	-0.09987	-0.9856
23	PC	-3.38089	1.900143	-2.31665	0.158983	-0.069	-0.23588	-0.1971	-0.11585
24	DPA	-2.96245	1.331037	-1.70189	0.280713	0.22183	-1.10686	-0.37429	-0.17033
25	CA	-2.96739	1.626564	-1.83297	0.287443	0.182035	-0.57191	0.013111	-0.27709

26	CC	-0.94236	2.813139	-1.82586	-0.92925	-0.2368	-0.26355	-0.97364	-0.6863
27	EC	-0.94236	2.813139	-1.82586	-0.92925	-0.2368	-0.26355	-0.97364	-0.6863
28	HT	-3.43864	1.394551	-1.73752	-0.20423	0.296465	-0.94031	-0.86765	0.006667
29	Rutin hydrate	4.755525	0.797689	-4.10561	-1.25962	-1.57885	-1.16111	-0.36028	0.214853
30	Vitamin C	-3.16688	-1.03724	-3.48814	0.268925	-0.59301	-0.33508	0.439129	0.145236
