# Supplementary material for: The SAR analysis of dietary polyphenols and their antagonistic effects on bortezomib at physiological concentrations
Source: Front Pharmacol. 2024 Jul 25;15:1403424. doi: 10.3389/fphar.2024.1403424 (PMC11306019; doi:10.3389/fphar.2024.1403424)
Supplement: Supplementary file 2 [file Presentation1.pdf]

The SAR analysis of Dietary Polyphenols and their Antagonistic Effects on Bortezomib at Physiological Concentrations

Tran Tran Thi Van, Hsun-Shuo Chang, Ho-Cheng Wu, Chung-Kuang Lu, Hui-Chi Huang, Michal Korinek, Hui-Hua Hsiao*, and Chia-Hung Yen*

Content:

1. Supplementary Figure 1
2. Supplementary Figure 2
3. Supplementary Figure 3
4. Supplementary Figure 4
5. Supplementary Figure 5
6. Supplementary Figure 6

Supplementary Figure 1

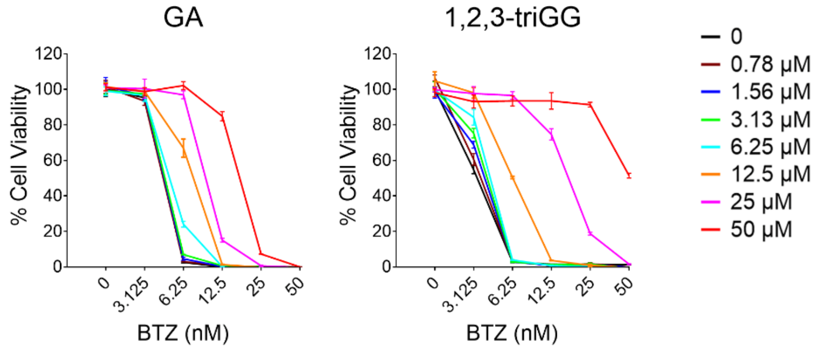

Supplementary Figure 1. The antagonistic effects against BTZ of GA and 1,2,3-triGG in RPMI 8226 cells. RPMI 8226 cells were treated with various concentrations of indicated polyphenols and BTZ both alone and in combination for 72 hours. Then, the cell viability assay was performed using resazurin, and the antagonistic index (AI) were calculated as described in the material and methods section and shown in Table 1. Data are presented as mean ± SD (n=3).

Supplementary Figure 2

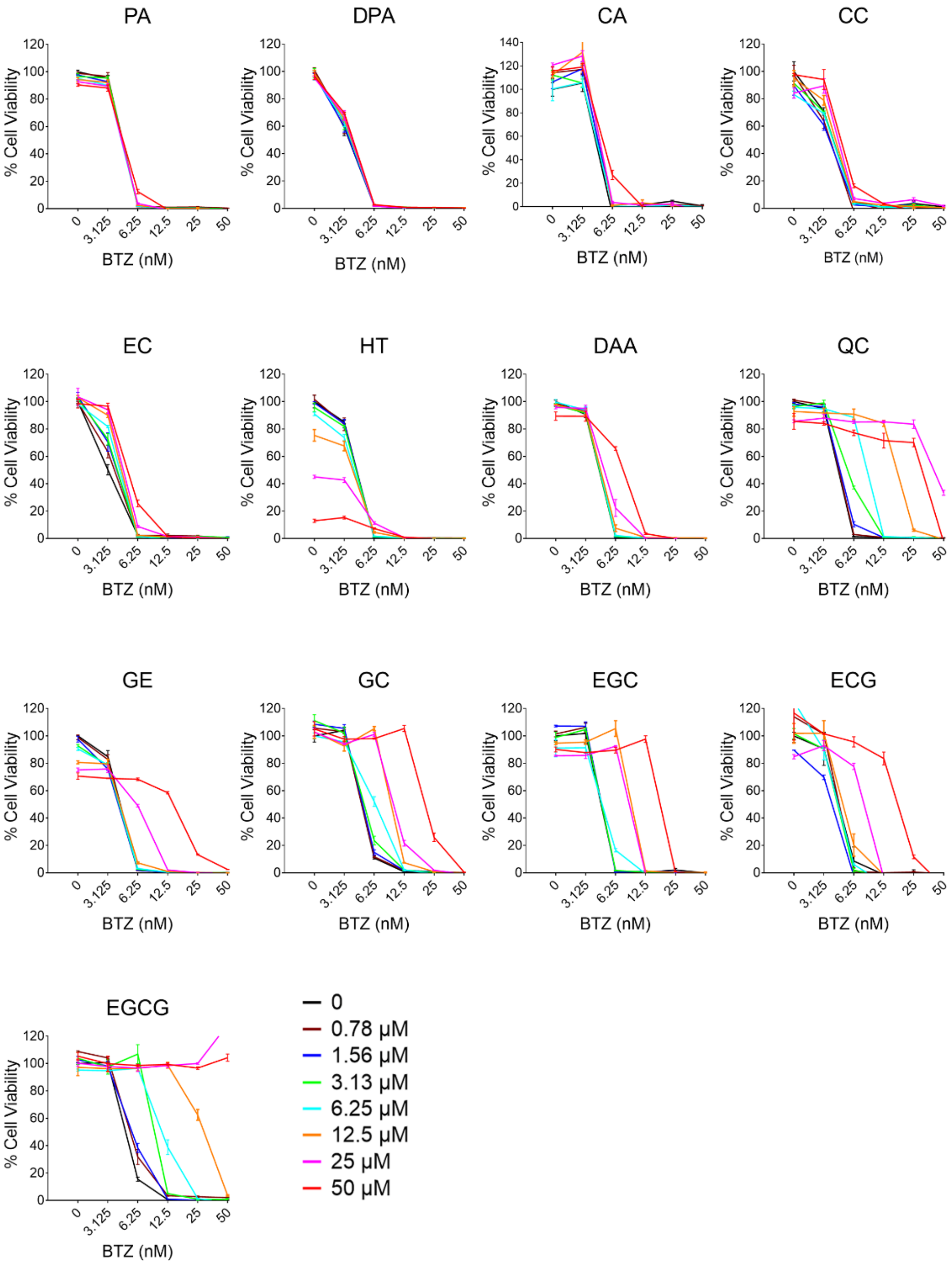

Supplementary Figure 2. The antagonistic effects against BTZ of dietary polyphenols in RPMI 8226 cells. RPMI 8226 cells were treated with various concentrations of indicated polyphenols and BTZ both alone and in combination for 72 hours. Then, the cell viability assay was performed using resazurin, and the antagonistic index (AI) were calculated as described in the material and methods section and shown in Table 1. Data are presented as mean ± SD (n=3).

Supplementary Figure 3

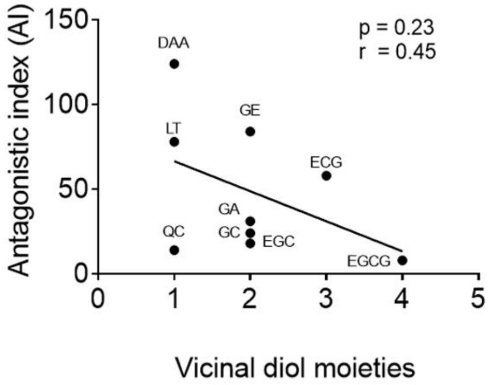

Supplementary Figure 3. The correlation between the number of vicinal diol moieties of nine dietary polyphenols and their AI value. Nine polyphenols used here include DAA, LT, QC, GE, GA, GC, EGC, ECG, and EGCG.

Supplementary Figure 4

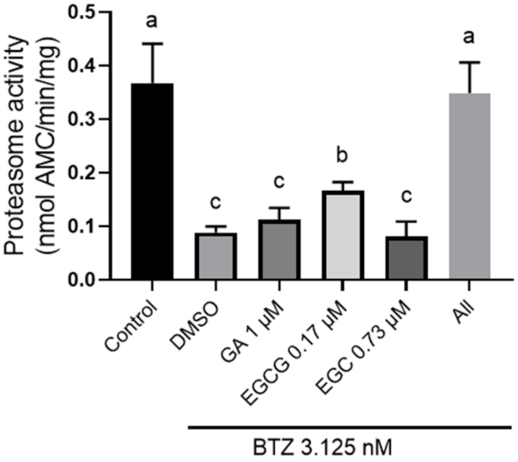

Supplementary Figure 4. Proteasome activity was determined from cells on the 8th day of treatment. The data represent the mean ± SD (n=3). Statistical analysis was performed using one-way ANOVA, followed by Tukey's multiple comparisons test where appropriate. Differences were considered significant when $P< 0.05$.

Supplementary Figure 5

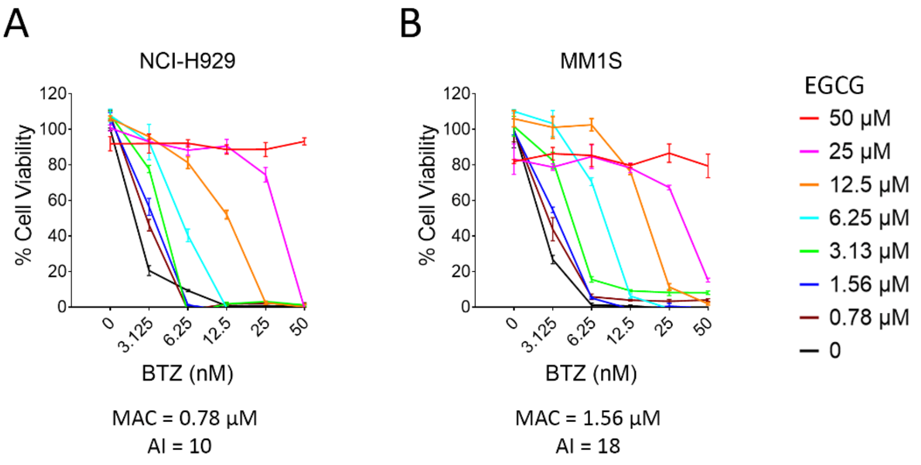

Supplementary Figure 5. EGCG showed antagonistic effects against BTZ in NCI-H929 (A) and MM1S cells (B). NCI-H929 and MM1S cells were treated with various concentrations of indicated EGCG and BTZ both alone and in combination for 72 hours. Then, the cell viability assay was performed using resazurin. Data are presented as mean ± SD (n=3).

Supplementary Figure 6

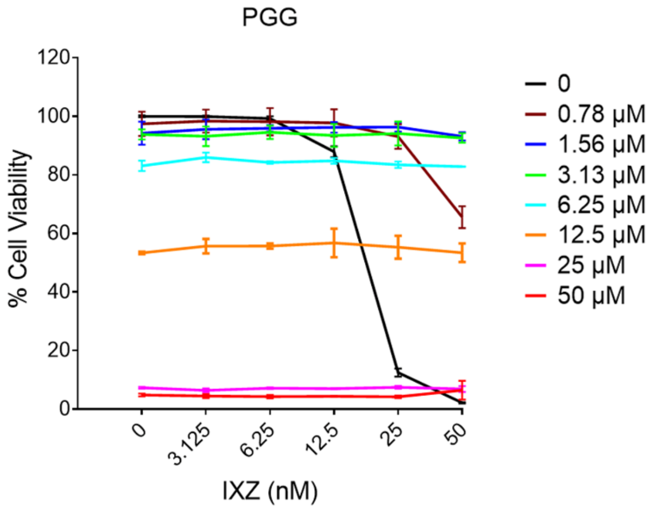

Supplementary Figure 6. 1,2,3,4,6-penta-*O*-galloyl-β-D-glucose (PGG) showed potent antagonistic effects against ixazomib (IXZ) in RPMI 8226 cells. RPMI 8226 cells were treated with various concentrations of indicated PGG and IXZ both alone and in combination for 72 hours. Then, the cell viability assay was performed using resazurin. Data are presented as mean ± SD (n=3).
